# Loss of humeral immunity in childhood cancer survivors not having undergone hematopoietic stem cell transplantation

**DOI:** 10.1002/cnr2.1907

**Published:** 2023-10-22

**Authors:** Benjamin Pearson, Michelle Pulley, Marcio Diniz, Nicole Baca, Fataneh Majlessipour

**Affiliations:** ^1^ Department of Health Studies University of Richmond Richmond Virginia USA; ^2^ Department of Pediatrics Cedars‐Sinai Medical Center Los Angeles California USA; ^3^ Biostatistics and Bioinformatics Research Center, Cedars‐Sinai Medical Center Los Angeles California USA; ^4^ Department of Pediatric Hematology and Oncology, Cedars‐Sinai Samuel Oschin Comprehensive Cancer Institute Los Angeles California USA

**Keywords:** and loss of immunity, childhood cancer survivors, vaccination

## Abstract

**Background:**

Data are limited and conflicting regarding loss of immunity in childhood cancer survivors who did not undergo hematopoietic stem cell transplantation. The purpose of this retrospective, single center study is to provide further data to help build unifying revaccination guidelines post‐chemotherapy in childhood cancer survivors not having undergone hematopoietic stem cell transplantation.

**Methods:**

This retrospective study included 28 childhood cancer survivors, 14 males and 14 females, whose treatment consisted of at least 3 months of chemotherapy and with confirmation of completing their primary vaccination series prior to therapy. The rate of vaccine titer seropositivity for cancer survivors was compared with the expected general population, based on long‐term studies of anti‐body persistence.

**Results:**

Decreased seropositivity for measles, mumps, rubella, varicella, tetanus, and hepatitis B was found in patients across all categories of malignancy compared with the general population. However, tetanus was not statistically significant. Results were more pronounced for those with hematological malignancies.

**Conclusions:**

This study indicates that pediatric cancer survivors, especially those with hematological malignancies, may have greater loss of protective antibodies from primary vaccinations. Further studies are needed to provide guidelines for revaccination of both hematologic malignancies and solid tumor childhood cancer survivors who did not undergo hematopoietic stem cell transplantation.

## 1 INTRODUCTION

Immunization is a fundamental part of pediatric healthcare and has significantly improved standards of international public health. Previous studies demonstrate intensive chemotherapy may cause notable decreases in vaccine‐specific antibody titers in childhood cancer survivors (CCS).^1^, ^2^, ^3^, ^4^, ^5^, ^6^ Adaptive immunity is divided into humoral immunity and cell‐mediated immunity that work together to produce antibodies with the help of T cells for a robust immune response to neutralize pathogens. After chemotherapy, cancer survivors suffer from acquired immunological defects making them vulnerable to vaccine‐preventable illnesses.^7^, ^8^ Chemotherapy regimens vary for different malignancies (i.e., type of antineoplastic agents, intensity, duration, schedule, etc.) with varying impacts on immune function. There is data citing impairment of humoral immunity demonstrated by reduced vaccine titers in survivors of acute lymphoblastic leukemia.^2^, ^7^, ^9^ Furthermore, there are small studies with conflicting results of immune functional outcomes following therapy for acute lymphoblastic leukemia.^2^, ^7^, ^8^, ^9^, ^10^

In CCS, the loss of protective antibodies poses a risk from vaccine‐preventable infections well after immune reconstitution.^11^ These potentially life‐threatening infections represent an avoidable source of morbidity and mortality and may create potential disease vectors that threaten public health initiatives.

While there are published guidelines for the revaccination of hematopoietic stem cell transplant (HSCT) patients, there are no unified guidelines for the revaccination of CCS not having undergone HSCT. The European Conference on Infections in Leukaemia published guidelines for hematological malignancies and the Italian Association Paediatric Haematology Oncology also has their own recommendations.^12^, ^13^, ^14^, ^15^ Vaccine‐specific titer data for CCS has been limited to studies with low sample sizes and conflicting conclusions.^16^, ^17^, ^18^, ^19^ This study's aim is to provide more information to address the knowledge gaps in immunological outcomes in childhood cancer therapy, which could help build standardized guidelines for this vulnerable population and provide opportunities for future research in the field of cancer survivorship.

## 2 METHODS

Cedars‐Sinai Medical Center (CSMC) Institutional Review Board approved this retrospective study and did not require consent due to being a minimal risk study The data from 28 patients, less than 21 years old, who underwent a minimum of 3 months of chemotherapy was collected from a list of CCS followed at CSMC (Los Angeles, CA) between January 1, 2010, and July 31, 2022, see Table 1. Of these patients 50% were male and 50% were female. Confirmation of primary vaccine series completion, either through available vaccination records or documentation in a physician note, was required to be included in the study. Exclusion criteria included relapsed disease, HSCT, or having received intravenous immunoglobulin (IVIG) within 6 months of the titer collection. All redrawn samples that occurred within 3 months of the original titer collection were grouped together. Serum antibody levels for measles, mumps, rubella, varicella, tetanus, and hepatitis B were measured between 1 month and 30 months after the conclusion of chemotherapy. These laboratory results were obtained from Clinical Laboratory Improvement Amendments (CLIA) certified laboratories. The cut off levels for the labs were normalized to positive or negative (if equivocal, considered negative) based upon the reference range from CLIA certified laboratory for each titer.

**TABLE 1:** Patient demographic, diagnosis, duration of treatment and time of titer drawn in proximity to the end of treatment.

Patient number	Sex	Race	Hispanic	Malignancy	Age at Diagnosis	Duration of therapy	Titer proximity to end of treatment
1	M	Other	No	B cell ALL	1 year, 3 months	38 months	2 years, 3 months
2	F	White	No	B cell ALL	1 year, 9 months	28 months	5 months
3	M	White	Yes	B cell ALL	1 year, 11 months	30 months	3 months
4	M	White	No	B cell ALL	2 years, 8 months	39 months	3 months
5	M	White	No	B cell ALL	3 years, 2 months	37 months	1 years, 11 months
6	M	White	No	B cell ALL	3 years, 2 months	37 months	4 months
7	M	White	Yes	B cell ALL	3 years, 6 months	39 months	5 months
8	F	White	No	B cell ALL	3 years, 11 months	27 months	1 years, 6 months
9	F	White	No	B cell ALL	5 years, 1 month	25 months	8 months
10	F	White	No	B cell ALL	5 years, 2 months	26 months	1 years, 3 months
11	F	Black	No	B cell ALL	6 years, 5 months	26 months	3 months
12	F	White	No	B cell ALL	6 years, 8 months	28 months	6 months
13	M	White	No	B cell ALL	7 years, 8 months	29 months	6 months
14	F	White	No	B cell ALL	9 years, 6 months	28 months	1 months
15	M	White	No	T cell ALL	1 year, 10 months	40 months	6 months
16	M	White	Yes	T cell ALL	8 years, 6 months	39 months	10 months
17	M	White	No	T cell ALL	11 years, 1 month	40 months	2 years, 6 months
18	M	Asian	No	T cell ALL	10 years, 4 months	39 months	5 months
19	F	White	No	Hodgkin lymphoma	7 years, 1 month	3 months	5 months
20	M	White	Yes	Non‐Hodgkin Lymphoma*	13 years, 5 months	24 months	9 months
21	F	While	No	Embryonal sarcoma of the liver	11 years, 0 months	6 months	7 months
22	M	Asian	No	Prostate embryonal rhabdomyosarcoma	13 years, 11 months	10 months	6 months
23	F	White	No	Cervical embryonal rhabdomyosarcoma	16 years, 11 months	6 months	8 months
24	F	White	No	Ewing sarcoma of right scapula	13 years, 5 months	9 months	5 months
25	M	White	No	Osteosarcoma of the right humerus	11 years, 5 months	8 months	4 months
26	F	White	No	Nephroblastoma	5 years, 9 months	7 months	5 months
27	F	White	Yes	Nephroblastoma	5 years, 11 months	5 months	10 months
28	F	Asian	No	Nephroblastoma	10 years, 5 months	4 months	11 months

*Note*: Mean age of diagnosis for all cancers: 7 years 10 months ± 4.5 years; mean age of diagnosis for hematological malignancies: 5 years 7.4 months ± 3.5 years. There was a 1:1 ratio of male to female patients. Type of cancers: 28.57% were solid tumors and 71.43% were hematologic malignancies.

* Non‐Hodgkin Lymphoma of the paranasal sinuses, subtype blastic plasmacytoid dendritic cell.

Abbreviations: ALL, acute lymphoblastic leukemia; F, female; M, male.

### 2.1 Serologic analysis

Protective levels of antibodies were determined by three entities through the span of the study Focus Diagnostics (San Juan Capistrano, CA), Quest Diagnostics (Secaucus, NJ), and CSMC (Los Angeles, CA), which are all Clinical Laboratory Improvement Amendments (CLIA) certified laboratories. The results of these titers were normalized to positive or negative (if equivocal, considered negative) values based on the metrics provided by the respective laboratories.

### 2.2 Statistical analysis

Data were entered into Microsoft Excel (Microsoft, WA) and statistics were computed within the software. As baseline titers were not available for all CCS subjects, the general population's immunity rate extracted from the literature was used as the control.^20^, ^21^, ^22^, ^23^, ^24^ Therefore, all *p*‐values listed are based upon the difference in this study cohort compared to this control. Binomial Exact tests and Wilson's Confidence Intervals were used to determine if the cohort's seropositivity was significantly different than the general population's rate. The general population's seropositivity rates, from individuals after completion of their primary vaccination series, were chosen from long‐term studies of antibody persistence. Seropositivity for measles, mumps, and rubella were set at 95%, 74%, and 100% respectively as found by Davidkin et al, 15–20 years after vaccination for a cohort of 180 subjects.^20^ Rubella seropositivity was adjusted to 99% to avoid divide by zero errors within Excel. Varicella seropositivity was set to 99% based on the results of Vessey et al and Watson et al^21^, ^24^ who took titers at 1 and 6 years, respectively. Although Vessey et al found 100% seropositivity at 1 year, a rate of 99% eliminates divide by zero errors in Excel. Tetanus seropositivity was set at 85.8% from a cohort of 1433 subjects as found by Borella‐Venturini et al^22^ for those who received <5 doses. Not all subjects received the 4th and 5th doses of the DTaP vaccine and therefore this seropositive value is more likely to mirror the study cohort. Hepatitis B had a greater literature base from which to choose a seropositivity rate. In following the CDC citations, seropositivity was set at 66% (15‐year mark) after following 1578 subjects as reported by McMahon et al.^23^

## 3 RESULTS

**FIGURE 1:**
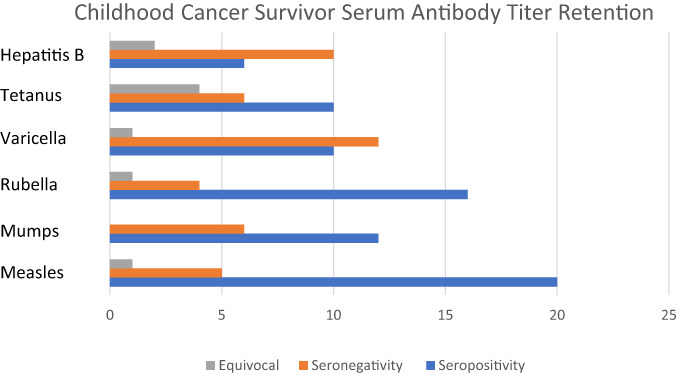
Childhood cancer survivor serum antibody titer retention. This figure includes all patients in the study, both hematological and solid tumor patients.

Compared to expected rates in the general population, CCS had lower rates of seropositivity for measles (20/26, 77%), mumps (12/18, 66%), rubella (16/21, 76.2%), varicella (10/23, 43.4%), tetanus (10/20, 50%), and hepatitis B (6/18, 33%). In both solid tumor and hematological malignancy subgroups, all values were significantly lower than literature rates of expected antibody retention, except for mumps CCS with hematological malignancies had the lowest rates of seropositivity with protective antibodies in only 70% (14/20) of measles, 61.5% (8/13) of mumps, 75% (12/16) of rubella, 31.25% (5/16) of varicella, 40% (6/15) of tetanus, and 23% (3/13) of hepatitis B titers. Figure 1 has a summary of CCS antibody retention rates and Table 2 has a summary of seronegative rates and statistical analysis.

**TABLE 2:** Seronegative of the general population compared to childhood cancer survivor (CCS) with all malignancies and CCS with hematological malignancies.

	General population	CCS all malignancies			CCS hematological malignancies		
	% w/o protection	% w/o protection	95% CI	*p* Value*	% w/o protection	95% CI	*p* Value*
Measles	5	23	11%–42%	*p* = .0002	30	14%–51.8%	*p* < .0001
Mumps	26	33	16%–56%	*p* = .16	41.6	19%–68%	*p* = .064
Rubella	1	23.8	10%–45%	*p* < .0001	25	10%–49%	*p* < .0001
VZV	1	56.5	36.8%–74%	*p* < .0001	68.75	44%–85.8%	*p* < .0001
Tetanus	14.2	50	29.9%–70%	*p* < .0001	60	35.7%–80%	*p* < .0001
Hepatitis B	33	66	43.7%–83.7%	*p* = .0007	76.9	49.7%–91.8%	*p* = .00019

* All *p*‐values listed are based upon the difference in this study cohort compared to expected controls from the literature.

## 4 DISCUSSION

Both cancer and cancer treatment have long‐term effects on various functions of the immune system. This study focused immune memory from primary vaccines in CCS. Data revealed a loss of immunity across the vaccine series suggesting immune memory is impaired by chemotherapy. Cancers involving the immune system, that is, hematological malignancies, resulted in a greater loss of immunity.

One study found seropositivity rates for measles, mumps, and rubella (MMR) of 95%, 74%, and 100%, respectively, 20 years after vaccination^13^ with other studies confirming these results.^25^, ^26^ While the general population tends to retain their protective antibodies, the CCS cohort retained immunity to measles, mumps, and rubella at a rate of 77%, 66%, and 76.2%, respectively. This effect was more dramatic in those with hematological malignancies, with 70%, 58.4%, and 75% retaining protection to measles, mumps, and rubella, representing a significant decrease in long‐term protection. Of note, mumps was the only titer that did not reach a *p* value <.05 in the study, which mirroring the lower antibody persistence of mumps in the control group. As per Davidkin et al, in long‐term studies of the general population noted that the proportion of low‐level (equivocal) antibodies was higher in mumps than in measles or rubella.^20^ Furthermore, the mumps component of the trivalent MMR vaccine is the weakest at inducing humoral immunity.^20^

As not all subjects had received the full five dose series for tetanus, the control seropositive rate of 85.2% using the Borella‐Venturin et al cohort with 1433 subjects who received <5 doses.^22^ Fifty percent (10/20) of our cohort retained seropositive titers to tetanus. The hematological malignancy group was more pronounced with only 40% (6/15) having protective titers.

Not all subjects received a booster of the varicella vaccine, but all received at least one. Therefore, the data from 1995, prior to the 2006 booster recommendations, was chosen as the control. Watson et al demonstrated 99% seropositivity 6 year after initial varicella vaccination.^21^ Across all malignancies, 43.4% of patients retained protective antibody levels with 31.25% of those treated for hematological malignancies retaining protective antibody levels.

Hepatitis B overall has the lowest rates of seropositivity after vaccination. In a 30‐year study of 1578 Alaska Natives vaccinated at age 6 months or older, 66% of individuals had protective surface antibody levels above 10 mIU/mL at 15 years.^23^ In contrast, only 33% (6/18) of our total cohort and 23% (3/13) of the hematological malignancies group retained protective antibody levels.

This retrospective study has several limitations. There were no pretreatment titers for most patients; thus, data was compared to expected vaccine seropositivity of the general population. By comparing post‐treatment titers to the expected general population vaccine seropositivity, we are assuming that the CCS in this study have seroconverted like the general population. It is possible that some malignancies, likely hematological malignancies, may impair the immune system and vaccine titers. It would be ideal to use the cohort pretreatment titers as the control to concretely identify CCS that have lost protective antibodies from therapy. Pediatric malignancies are rare and the cohort was limited to one institution. Furthermore, the chemotherapy regimens varied because this study included all CCS receiving 3 months of chemotherapy who did not relapse nor require a HSCT. The authors acknowledge that the small sample size and the cohort restriction to a single center limit the potential generalizability to all CCS.

Despite limitations, these results bolster the idea that CCS likely need to be revaccinated after completing treatment. CCS, especially hematological malignancies patients, appear to have lower seropositivity rates when compared with the general population. After chemotherapy, it may take months to years for complete immune reconstitution^11^, ^16^, ^17^; however, it is unknown how much immune recovery is essential for patients to seroconvert after revaccination. Future studies should include pre‐treatment vaccine titers and post‐treatment titers in a statistically significant number of subjects for both hematological and solid tumors. Performing testing to assess immune recovery, such as lymphocyte function, may help determine the optimal time for re‐vaccination.

These findings demonstrate a statistically significant loss of immunity to measles, rubella, varicella, tetanus, and hepatitis B in CCS; however, no statistically significant difference in mumps antibody persistence. Future prospective studies are needed to build comprehensive guidelines for CCS who have not undergone HSCT.

## AUTHOR CONTRIBUTIONS

Fataneh Majlessipour conceived the study. Fataneh Majlessipour and Nicole Baca contributed to sample acquisition. All authors contributed to study design, data analysis, interpretation. Benjamin Pearson, Michelle Pulley, Nicole Baca, and Fataneh Majlessipour contributed to manuscript writing and all authors had final approval of the manuscript.

## CONFLICT OF INTEREST STATEMENT

The authors have stated explicitly that there are no conflicts of interest in connection with this article.

## ETHICS STATEMENT

Cedars‐Sinai Medical Center (CSMC) Institutional Review Board approved this retrospective study and did not require consent due to being a minimal risk study.

## Data Availability

The data that support the findings of this study are available from the corresponding author upon reasonable request.
